# New Prenylated Aeruginosin, Microphycin, Anabaenopeptin and Micropeptin Analogues from a *Microcystis* Bloom Material Collected in Kibbutz Kfar Blum, Israel

**DOI:** 10.3390/md13042347

**Published:** 2015-04-15

**Authors:** Shira Elkobi-Peer, Shmuel Carmeli

**Affiliations:** Raymond and Beverly Sackler Faculty of Exact Sciences, Raymond and Beverly Sackler School of Chemistry, Tel Aviv University, Ramat Aviv, Tel Aviv 69978, Israel; E-Mail: shirapeer@gmail.com

**Keywords:** *Microcystis*, aeruginosin, anabaenopeptin, micropeptin, microphycin, cyclic peptide, protease inhibitor, nuclear magnetic resonance, mass spectrometry

## Abstract

Thirteen new and eighteen known natural products were isolated from a bloom material of an assembly of various *Microcystis* spp. collected in November, 2008, from a commercial fishpond near Kibbutz Kfar Blum, the Jordan Valley, Israel. The new natural products included the prenylated aeruginosin KB676 (**1**), microphycin KB921 (**2**), anabaenopeptins KB906 (**3**) and KB899 (**4**) and micropeptins KB928 (**5**), KB956 (**6**), KB970A (**7**), KB970B (**8**), KB984 (**9**), KB970C (**10**), KB1048 (**11**), KB992 (**12**) and KB1046 (**13**). Their structures were elucidated primarily by interpretation of their 1D and 2D nuclear magnetic resonance spectra and high-resolution mass spectrometry. Marfey’s and chiral-phase high performance liquid chromatography methods were used to determine the absolute configurations of their chiral centers. Aeruginosin KB676 (**1**) contains the rare (2*S*,3a*S*,6*S*,7a*S*)-Choi and is the first prenylated aeruginosin derivative described in the literature. Compounds **1** and **5**–**11** inhibited trypsin with sub-μM IC_50_s, while Compounds **11**–**13** inhibited chymotrypsin with sub-μM IC_50_s. The structures and biological activities of the new natural products and our procedures of dereplication are described.

## 1. Introduction

Cyanobacterial water blooms are initiated from dormant cells under appropriate environmental conditions, which develop into a stable population of different biomass intensities and toxin content, which eventually collapses, disintegrates and discharges toxins to the water environment [1]. These blooms are frequently described as a single-species phenomenon. However, recent studies suggest that many of these blooms are composed of complex populations of various strains of one or more species of the same genus, as well as associated bacteria, and the community structure varies in time and space [2,3]. The natural products that are biosynthesized by the community members most probably govern the variation of the community in space and time [4,5]. A large volume of research was devoted to the toxins that are produced by cyanobacterial blooms and to the toxic action of these toxins; microcystins, anatoxins, saxitoxins and cylindrospermopsins [6,7]. However, recent studies have suggested that microcystins are involved in quorum sensing and might be produced to manage cyanobacterial colonies in the environment [8] and not necessarily produced for their toxic effects.

The protease inhibitors that are produced by microcystin-producing genera of cyanobacteria, *i.e.*, micropeptins, aeruginosins, anabaenopeptins, microginins and microviridins, have been shown to affect the normal life cycle of organisms that interact with cyanobacteria in the environment [9]. These secondary metabolites usually appear in the bloom in relatively high concentrations as an array of structurally-related compounds, and their ecological role is still not fully understood [10].

As part of our continuous interest in the pharmacological properties and the ecological role of cyanobacterial natural products [11], we chemically investigated the extracts of a bloom material composed of an assembly of *Microcystis* spp. collected in November, 2008, from a commercial fishpond near Kibbutz Kfar Blum, the Jordan Valley, Israel. Thirteen new natural products, aeruginosin KB676 (**1**), microphycin KB921 (**2**), anabaenopeptins KB906 (**3**) and KB899 (**4**) and micropeptins KB928 (**5**), KB956 (**6**), KB970A (**7**), KB970B (**8**), KB984 (**9**), KB970C (**10**), KB1048 (**11**), KB992 (**12**) and KB1046 (**13**), and eighteen known natural products were isolated from this bloom material. The known natural products were aeruginazoles A [12] and DA1304 [13], aeruginosins 298B [14] and DA495A [15], anabaenopeptins G [16], H [16], 908 [17], 915 [17], HU892 [18] and MM913 [19], cyanopeptolins S [20] and SS [21], ichthyopeptin A [22], microcystin-LR [23], micropeptins HM978 [24], LH920 [25] and LH1021 [25] and oscillamide C [26] (Supplementary Figures S1 and S2). The structural elucidation and biological activity of the thirteen new compounds are described below.

## 2. Results and Discussion

Thirty-one natural products were isolated from a 70% aqueous methanol extract of bloom material collected from a fishpond of the Kibbutz Kfar Blum. The compounds were separated through fractionation by reversed-phase C_18_ open column, size exclusion chromatography and purification on various reversed-phase high performance liquid chromatography (HPLC) columns. The fractionation process was guided by the serine protease inhibition assay. Dereplication and verification of the purity of the isolated natural products was achieved by running liquid chromatography mass spectrometry (LCMS) and nuclear magnetic resonance (NMR) spectra on each one of the isolated compounds.

### 2.1. Structural Elucidation of Aeruginosin KB676

**Figure 1:**
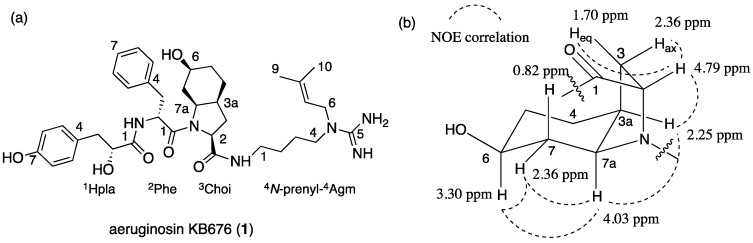
(**a**) The structure of aeruginosin KB676 (**1**); and (**b**) chemical shifts (in ppm) and NOE correlations of the l-6-epiChoi moiety of **1**.

Aeruginosin KB676 (**1**, Figure 1a) was isolated as a glassy material that presented a high-resolution electrospray ionization mass spectrometry (HR ESI MS) protonated molecular ion at *m*/*z* 677.4031 corresponding to a molecular formula of C_37_H_53_N_6_O_6_ and 15 degrees of unsaturation. However, its ^1^H and ^13^C NMR spectra in DMSO-*d*_6_ (Supplementary Figures S3 and S4) seemed much more complicated than the expected spectra for 53 protons and 37 carbons. Careful examination of these spectra revealed that many of the signals appeared as pairs of signals with slightly different chemical shifts and integration of 55:45, while some presented doubled integration and a single chemical shift.

A full assignment of the NMR data (Table 1 for the major rotamer, and Supplementary Table S1 for the minor rotamer) by interpretation of the correlations from homo-nuclear (COSY, TOCSY and ROESY) and hetero-nuclear (HSQC and HMBC) 2D NMR experiments revealed that **1** contained pairs of the following acid units; *p*-hydroxyphenyl lactic acid (Hpla), Phe, 2-carboxy-6-hydroxy-octahydro-indole (Choi), characteristic of the aeruginosins, and ^4^*N*-prenyl-agmatine [15]. The Choi moiety was assigned as follows: COSY and TOCSY correlations connected H-2 through H-3ax and H-3eq to H-3a, while H-3a presented COSY correlations with H_2_-4 and H-7a. The small difference in the chemical shifts of H_2_-4 and H-5eq did not allow the unequivocal connection of H_2_-4 and H_2_-5. However, COSY correlations of H-7a, through H-7ax, H-7eq, H-6, 6-OH, to H-5ax and H-5eq, allowed the connection of all of the protons of the Choi moiety. TOCSY correlations connected H-3a with H-5ax and closed the six-membered ring of the Choi moiety. The carbons of the Choi moiety were assigned by the HSQC correlations, except for C-1 (δ_C_ 172.4) that, in the case of the major rotamer, did not show a correlation with the Choi protons, but showed a correlation with Agm-1-NH. It was assigned on the basis of the correlations of the minor rotamer C-1 (δ_C_ 171.6) with Choi-H-2 and Agm-1-NH. NOE correlations from a ROESY experiment (Figure 1b) established that H-2, H-3a, H-6 and H-7a pointed to the same face of the Choi moiety. A comparison of the protons’ and carbons’ chemical shifts and the NOE correlations of the Choi moiety protons of **1** with those of aeruginosin KT608 ((2*R*,3a*R*,6*R*,7a*R*)-Choi (d-3a,7a-*diepi*Choi)) [27], aeruginosin DA495A ((2*S*,3a*S*,6*S*,7a*S*)-Choi (l-6-*epi*Choi)) [15] and aeruginosin DA642A ((2*S*,3a*S*,6*R*,7a*S*)-Choi (l-Choi)) [15] revealed that it matched those of aeruginosins KT608 and DA495A and, thus, displayed all S* relative configurations. A comparison of the retention times of the 1-Choi-2,4-dinitrophenyl-5-l-alanine amide (Choi-l-DAA) derivative (Marfey’s analysis [28]) of **1** with those of authentic samples of (2*R*,3a*R*,6*R*,7a*R*)-Choi-l-DAA, (2*S*,3a*S*,6*S*,7a*S*)-Choi-l-DAA and (2*S*,3a*S*,6*R*,7a*S*)-Choi-l-DAA revealed that **1** contained ((2*S*,3a*S*,6*S*,7a*S*)-Choi (l-6-*epi*Choi)), similar to that of aeruginosin DA495A [15], which was isolated with it from the same extract.

**Table 1:** NMR data of the major rotamer of aeruginosin KB676 (**1**) in DMSO-*d*_6_^a^.

Position	δ_C_ mult. ^b^	δ_H_ mult. ^b^ *J* in Hz	HMBC Correlations ^c^	NOE Correlations ^d^
^1^Hpla				
1	173.5 qC	-		
2	72.4 CH	3.87 m		Hpla-3,3′,5,5′; Phe-5,5′,6,6′,*N*H
3	40.0 CH_2_	2.60 dd (14.0,3.5)	Hpla-1,2,4,5,5′	Hpla-2,3′,5,5′
		2.27 dd (14.0,8.5)	Hpla-1,2,4,5,5′	Hpla-2,3,5,5′
4	128.6 qC	-		
5,5′	130.3 CH	6.85 d (8.3)	Hpla-3,6,6′,7	Hpla-2,3,3′,6,6′
6,6′	115.0 CH	6.59 d (8.3)	Hpla-4,7	Hpla-5,5',7-*O*H
7	155.8 qC	-		
2-*O*H	-	5.31 brs		
7-*O*H	-	9.09 s	Hpla-7	Hpla-6,6′
^2^Phe				
1	170.0 qC	-		
2	51.4 CH	4.26 q (6.0)	Phe-1,3	Phe-3,5,5′,*N*H; Choi-2,3
3	36.6 CH_2_	2.80 m	Phe-1,4	Phe-2,5,5′,*N*H
4	138.2 qC	-		
5,5′	129.3 CH	7.10 m	Phe-3,7	Phe-2,3,6,6′; Hpla-2
6,6′	128.2 CH	7.22 m	Phe-4	Phe-3,5,5′,7; Hpla-2
7	126.4 CH	7.16 m	Phe-5	Phe-6,6′
*N*H	-	7.76 d (8.0)	Hpla-1; Phe-2	Phe-2,3; Hpla-2
^3^Choi				
1	172.4 qC	-		
2	59.7 CH	4.79 d (9)	Choi-3	Choi-3,3′,3a; Phe-2; Agm-1-*N*H
3 (ax) (eq)	32.8 CH_2_	2.36 m	Choi-7a	Choi-2,3′,3a; Phe-2
		1.70 m		Choi-2,3,3a; Phe-2
3a	32.2 CH	2.25 m		Choi-2,3,3′,4,7a
4	22.8 CH_2_	1.59 m, 2H		Choi-3a,5,6,7a
5 (eq) (ax)	29.9 CH_2_	1.57 m		Choi-4,5′,6
		1.22 m		Choi-4,5,7ax
6	67.0 CH	3.30 m		Choi-4,5,7eq,7a
6-OH	-	4.55 brs		
7 (eq) (ax)	36.2 CH_2_	2.36 m	Choi-3a,6,7a	Choi-4,6,7′,7a; Phe-2
		0.82 m		Choi-5ax,7eq
7a	56.8 CH	4.03 m		Choi-3a,5,6,7eq
^4^*N*-prenyl-^4^Agm				
1	38.6 CH_2_	3.16 m	Choi-1	Agm-1′,2,3,6,7,1-*N*H
		3.05 m	Choi-1, Agm-2,3	Agm-1,2,3,1-*N*H
2	26.4 CH_2_	1.40 m	Agm-1,3	Agm-1,1′,3,4,6,1-*N*H
3	24.7 CH_2_	1.48 m	Agm-1,2	Agm-1,1′,2,4,6,7,1-*N*H
4	47.3 CH_2_	3.16 m	Agm-2,3,5,6	Agm-2,3,6,7,1-*N*H
5	155.8 qC	-		
6	45.9 CH_2_	3.87 brd (6.0)	Agm-4,5,7,8	Agm-1,2,3,4,7,9,10
8	137.1 qC	-		
9	25.7 CH3	1.70 brs	Agm-7,8	Agm-6,7
10	18.0 CH3	1.64 brs	Agm-7,8	Agm-6,1-*N*H
1-*N*H	-	8.23 t (5.5)	Choi-1	Agm-1,1′,2,3,4,10; Choi-2
5-*N*H,*N*H_2_	-	7.25 brm		

^a^ One hundred twenty five megahertz for carbons and 500 MHz for protons; ^b^ multiplicity: qC, quaternary carbon; CH, methane carbon; CH_2_, methylene carbon; CH_3_, methyl carbon; assigned from HSQC experiment; ^c^ determined from the HMBC experiment, ^n^*J*CH = 8 Hz, recycle time 1 s; ^d^ selected NOEs from the ROESY experiment.

The structure of the unique ^4^*N*-prenyl-agmatine was established based on COSY correlations of 1-NH through CH_2_-4 and HMBC correlations of the guanidine carbon (C-5, δ_C_ 155.8) with H_2_-4 and H_2_-6. The structure of the prenyl substituent was established by the COSY correlations of H_2_-6 through H_3_-9 and H_3_-10 and the HMBC correlations of H_2_-6, H_3_-9 and H_3_-10 with the double-bond carbons (δ_C_ 118.6, CH and 137.1 qC). The four units were assembled to the full planar structure by an HMBC correlation of ^1^Hpla-CO with ^2^Phe-NH, NOE correlations of ^1^Hpla-H-2 with ^2^Phe-NH, of ^2^Phe-H-2 and ^3^Choi-H-2 and of ^3^Choi-H-2 with ^4^Agm-1-NH. The NOE correlations of ^2^Phe-H-2 and ^3^Choi-H-2 in the major rotamer established it as the *cis* rotamer, while the NOE of ^2^Phe-H-2 and ^3^Choi-H-7a in the minor rotamer verified it as the *trans* rotamer. Marfey’s analysis [28] established the configuration of the Phe residue as d, while chiral HPLC established the configuration of Hpla residue as D. Based on these arguments, the structure of aeruginosin KB676 was established as **1**.

Several *N*-, *O*- and *C*-prenylated peptide-derived metabolites have been described from cyanobacteria. Kawaguchipeptin A [29] contains two *C*-prenylated tryptophan units within its cyclic undecapeptide structure. Prenylagaramides A and B are *O*-prenylated cyanobactins isolated from *Oscillatoria* (*Planktothrix*) *agardhii* [30]. *N*-prenylated peptides are more common in cyanobacterial extracts as exemplified by aeruginoguanidines 98-A-98-C [31], stictamides A–C [32] and microguanidine AL772 [33]. Aeruginosin KB676 (**1**) is the first prenylated aeruginosin derivative. It is related in structure to aeruginosin KT608B (IC_50_ (against trypsin) 1.3 μM) [27], except for the stereochemistry of the Choi, which resembled that of aeruginosin DA495A [15], and the agmatine prenylation. The reason for the biosynthesis of different isomers of the Choi moiety and prenylation of agmatine remains unclear.

### 2.2. Structural Elucidation of Microphycin KB921

**Figure 2:**
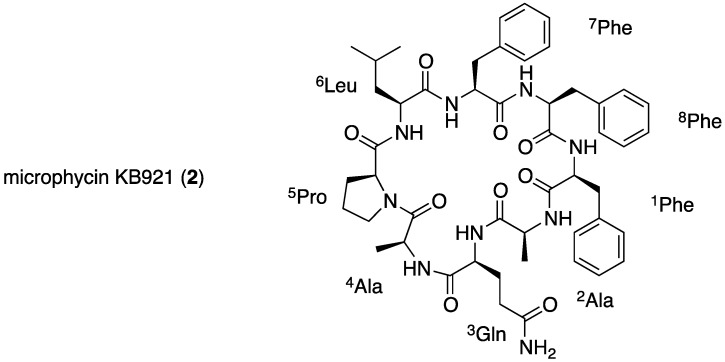
The structure of microphycin KB921 (**2**).

**Figure 3:**
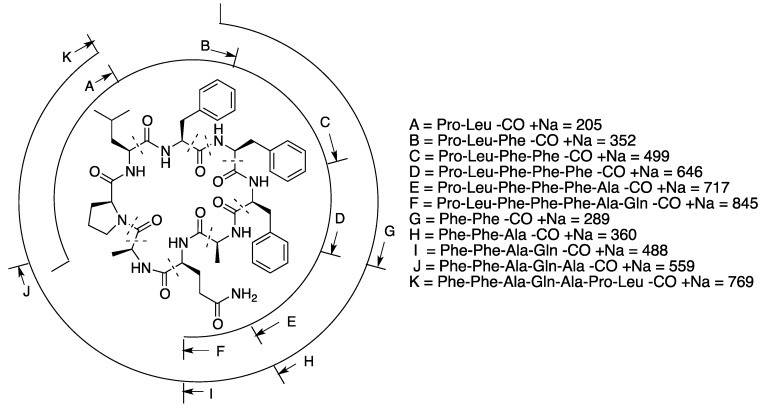
Sequence assignment of **2** by fragmentation of the parent ion of the ESI MS/MS quasi-molecular sodium ion.

Microphycin KB921 (**2**, Figure 2) was isolated as a transparent solid material presenting an HR ESI MS quasi-molecular ion ([M + Na]^+^) at *m*/*z* 944.4650, which corresponded to a molecular formula of C_49_H_63_N_9_NaO_9_. The molecular formula of **2** and its NMR spectra in DMSO-*d*_6_ (Table 2) pointed to its peptidic nature. The 23 degrees of unsaturation suggested, beside the nine carbonyls, three phenyl rings and a pyrrolidine ring, a cyclic peptide structure. A full assignment of the ^1^H and ^13^C NMR spectra of **2** in DMSO-*d*_6_ (Supplementary Table S2) established the structures of three Phe, two Ala, one Pro, one Gln and one Leu moieties. HMBC correlations (Supplementary Table S2) allowed the construction of two short amino acid sequences: ^5^Pro-^6^Leu-^7^Phe-^8^Phe-^1^Phe and ^2^Ala-^3^Gln. NOE correlations from a ROESY experiment (Supplementary Table S2) enabled the connection of ^1^Phe-H-2 with ^2^Ala-NH and ^4^Ala-NH with ^5^Pro-H-5 extending the amino acid sequence to: ^4^Ala-^5^Pro-^6^Leu-^7^Phe-^8^Phe-^1^Phe-^2^Ala-^3^Gln. However, the closure of the macrocyclic ring was evident only from the molecular weight of **2**. To support this assumption, an MS/MS measurement was performed for **2**, and some of the indicative data supporting the closure of the cycle between ^3^Gln-CO and ^4^Ala-NH are presented in Figure 3. Applying Marfey’s method [28] to the amino acid derivatives of **2** established all amino acids as being of the l-configuration. Based on these arguments, the structure of **2** was assigned to microphycin KB921.

**Table 2:** ^1^H and ^13^C NMR data of Compounds **2**–**4** in DMSO-*d*_6_.

Compound							
	2			3		4	
Position	δ_C_^a^	δ_H_^b^	Position	δ_C_^a^	δ_H_^b^	δ_C_^c^	δ_H_^d^
^1^Phe			^1^Ile				
1	171.1 qC		1	170.7 qC	-	170.7 qC	-
2	52.9 CH	4.91 brdd	2	58.1 CH	4.19 m	58.1 CH	4.15 m
3	39.0 CH_2_	3.38 m	3	36.1 CH	1.97 m	35.9 CH	1.98 m
		2.62 t					
4	137.7 qC	-	4	24.2 CH_2_	1.31 m	24.2 CH_2_	1.28 m
					0.98 m		0.93 m
5,5′	129.7 CH	7.31 m	5	11.6 CH_3_	0.75 t	11.4 CH_3_	0.75 t
	128.2 CH	7.27 m	6	16.1 CH_3_	0.77 d	16.1 CH_3_	0.76 d
7	126.5 CH	7.20 m	*N*H	-	8.10 d	-	8.17 d
*N*H		7.27 m	^2^*N*MeHty				
^2^Ala			1	169.5 qC	-	169.4 qC	-
1	173.6 qC	-	2	59.5 CH	4.66 t	59.5 CH	4.58 t
2	52.6 CH	3.84 dq	3	30.9 CH_2_	2.03 m	30.8 CH_2_	1.98 m
					1.70 m		1.70 m
3	17.0 CH_3_	1.31 d	4	31.6 CH_2_	2.32 m	31.4 CH_2_	2.31 m
					2.23 m		2.27 m
*N*H		8.96 m	5	131.6 qC	-	131.6 qC	-
^3^Gln			6,6′	129.1 CH	6.95 d	129.0 CH	6.95 d
1	174.0 qC	-	7,7′	115.4 CH	6.66 d	115.3 CH	6.66 d
2	53.1 CH	4.08 dt	8	155.7 qC	-	155.7 qC	-
3	29.7 CH_2_	2.12 m	*N*Me	28.8 CH_3_	2.58 s	28.6 CH_3_	2.57 s
		1.84 m					
4	31.8 CH_2_	2.14 m	^3^Hph				
5	174.3 qC	-	1	172.4 qC	-	173.2 qC	-
*N*H	-	8.18 d	2	48.6 CH	4.71 m	48.1 CH	4.69 m
*N*H_2_	-	6.80 s	3	33.2 CH_2_	2.00 m	33.2 CH_2_	2.02 m
		7.33 s			1.78 m		1.75 m
^4^Ala			4	31.7 CH_2_	2.81 m	31.6 CH_2_	2.81 m
					2.66 m		2.66 m
1	171.9 qC	-	5	141.3 qC	-	141.2 qC	
2	47.2 CH	4.58 dq	6,6’	128.6 CH	7.25 d	128.6 CH	7.25 m
3	16.4 CH_3_	1.21 d	7,7’	128.6 CH	7.23 t	128.5 CH	7.23 m
*N*H	-	7.07 m	8	126.4 CH	7.18 t	126.3 CH	7.18 m
^5^Pro			*N*H	-	8.93 d	-	8.98 d
1	174.8 qC	-	^4^Ile/Val				
2	62.8 CH	4.31 t	1	173.1 qC	-	172.9 qC	-
3	29.2 CH_2_	2.15 dd	2	55.6 CH	4.21 t	58.1 CH	3.94 t
		1.67 m					
4	24.6 CH_2_	1.85 m	3	36.4 CH	1.80 m	30.1 CH	1.88 m
		1.47 m					
5	47.3 CH_2_	3.54 m	4	25.7 CH_2_	1.34 m	19.1 CH_3_	0.88 d
		3.38 m			1.12 m		
^6^Leu			5	11.8 CH_3_	0.83 t	18.7 CH_3_	0.86 d
1	171.0 qC	-	6	14.5 CH_3_	0.78 d	-	-
2	53.8 CH	3.67 m	*N*H	-	6.70 d	-	6.78 d
3	38.6 CH_2_	1.30 m	^5^Lys				
		1.11 m					
4	24.3 CH	1.33 m	1	172.4 qC	-	172.4 qC	-
5	22.6 CH_3_	0.79 d	2	55.0 CH	3.88 ddd	54.9 CH	3.85 ddd
6	21.6 CH_3_	0.67 d	3	30.9 CH_2_	1.61 m	31.1 CH_2_	1.61 m
*N*H	-	8.16 d	4	20.6 CH_2_	1.30 m	20.6 CH_2_	1.39 m
					1.24 m		1.32 m
^7^Phe			5	28.3 CH_2_	1.38 m	28.2 CH_2_	1.40 m
1	170.4 qC	-	6	38.2 CH_2_	3.48 m	38.3 CH_2_	3.43 m
					2.78 m		
2	53.2 CH	4.32 brdd	α*-N*H	-	6.51 d	-	2.81 m
3	36.9 CH_2_	3.47 m	*ε-N*H	-	7.13 m	-	7.07 m
		2.73 t					
4	138.6 qC	-	^6^Arg/Tyr				
5,5′	129.2 CH	7.14 m	1	174.3 qC	-	173.9 qC	-
6,6′	128.2 CH	7.22 m	2	52.1 CH	4.09 dt	54.2 CH	4.26 m
7	126.4 CH	7.17 m	3	29.6 CH_2_	1.69 m	36.9 CH_2_	2.86 m
					1.52 m		2.75 m
*N*H	-	7.78 d	4	25.2 CH_2_	1.46 m	127.4 qC	-
^8^Phe			5	40.5 CH_2_	3.09 m	130.3 CH	6.95 d
1	169.5 qC		6	156.9 qC	-	115.2 CH	6.64 d
2	56.9 CH	3.65 m	7	-	-	156.0 qC	-
3	32.6 CH_2_	3.22 m	α*-N*H	-	6.42 d	-	6.19 d
4	139.5 qC	-	δ *-N*H/OH	-	7.50 brm	-	9.21 s
5,5′	129.0 CH	7.07 m	*N*H/*N*H_2_	-	6.65 brm		
					7.30 brm		
6,6′	128.4 CH	7.20 m	CO	157.5 qC	-	157.2 qC	
7	126.2 CH	7.18 m					
*N*H	-	7.57 d					

^a^ One hundred twenty five megahertz; multiplicity: qC, quaternary carbon; CH, methane carbon; CH_2_, methylene carbon; CH_3_, methyl carbon; assigned from the HSQC experiment; ^b^ 500 MHz; ^c^ 100 MHz; ^d^ 400 MHz.

Microphycin KB921 (**2**) most probably belongs to the growing family of cyanobactins that are synthesized ribosomally. This family includes cyclic peptides, such as prenylagaramides A and B [30], planktocyclin [34] and anacyclamide [35], but their role in cyanobacteria is yet unknown.

### 2.3. Structural Elucidation of Anabaenopeptins KB906 and KB899

**Figure 4:**
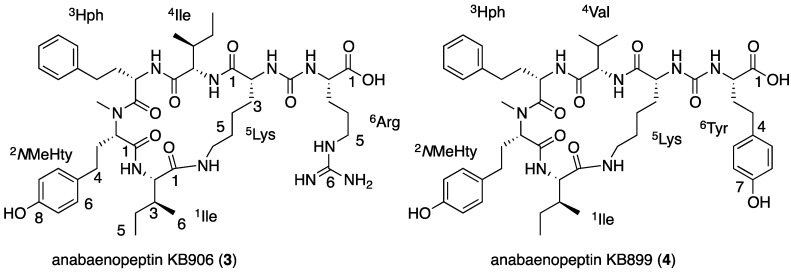
The structures of anabaenopeptins KB906 (**3**) and KB899 (**4**).

Compounds **3** and **4** (Figure 4) were identified as new anabaenopeptins based on their mass weights and the characteristic signals of the urea bridge in the proton and carbon NMR spectra in DMSO-*d*_6_, two amide doublet signals between 6 and 7 ppm in the proton spectrum and a carbonyl signal between 157 and 158 ppm in the carbon spectrum [16].

Anabaenopeptin KB906 (**3**) was isolated as a white solid material that presented an HR ESI MS protonated molecular ion at *m*/*z* 907.5417 and a molecular formula of C_46_H_71_N_10_O_9_. The ^1^H NMR spectrum of **3** in DMSO-*d*_6_ (Table 2) presented in the lower field proton signals characteristic of phenol-OH, seven secondary amide protons, a phenyl and a *para* substituted phenol ring. Signals of six methines, two methylenes and a methyl next to electron withdrawing atoms appeared in mid-spectrum, while two doublet and two triplet methyl signals were evident among other signals in the aliphatic region of the ^1^H NMR spectrum. In the ^13^C NMR spectrum (Table 2), **3** presented six acid/amide carbonyl signals around 170 ppm, three quaternary carbon signals around 156 ppm and additional two quaternary and five methine carbon signals at the aromatic region, six methine carbons next to electron withdrawing groups in mid-spectrum and a handful of signals in the upper field of the spectrum. The assignment of the proton and carbon signals to the following amino acid building blocks—2 × Ile, homophenylalanine (Hph), *N*-methylhomotyrosine (*N*MeHty), Arg and ε-substituted-Lys—was achieved by interpretation of the data from the 2D NMR spectra (Supplementary Table S3). These building blocks were assembled into the planar structure of **3** through the HMBC correlations of ^2^*N*MeHty-CO with the amide proton of ^1^Ile, of ^3^Hph-CO with ^2^*N*MeHty-H-2, of ^5^Lys-CO with the amide proton of ^4^Ile, of ^1^Ile-CO with the ε-amide proton of ^4^Lys- and of the uryl-CO with the amide proton of ^6^Arg and ^5^Lys-H-2 and the NOE correlations of ^1^Ile-NH and ^2^*N*MeHty-H-2, of ^4^Ile-H-2 with ^3^Hph-NH, of ^5^Lys-ε-NH with ^1^Ile-H-2 and of ^5^Lys-α-NH with ^6^Arg-α-NH. The application of Marfey’s method [28] to the hydrolysate of **3** established Ile, Hph, *N*MeHty and Arg as l-configuration and Lys as d-configuration. Based on these arguments, the structure of anabaenopeptin KB906 was assigned as **3** (Figure 4).

**Figure 5:**
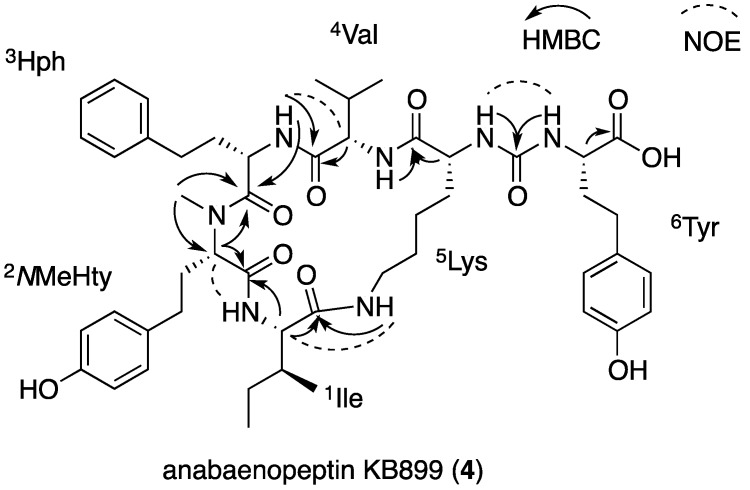
HMBC and NOE correlations that established the amino acid sequence of **4**.

Anabaenopeptin KB899 (**4**) presented comparable NMR spectra to those of **3** (Table 2) and an HR ESI MS quasi-molecular ion ([M + Na]^+^) at *m/z* 922.4693 corresponding to the molecular formula C_48_H_65_N_7_NaO_10_. The full assignment of its NMR data (Supplementary Table S4) indicated that it shared ^1^Ile, ^2^Hph, ^3^*N*MeHty and ^5^Lys with **3**, but contained ^4^Val instead of ^4^Ile and ^6^Tyr instead of ^6^Arg. The HMBC and NOE correlations that assisted in assembling the planar structure of **4** are presented in Figure 5. Marfey’s analysis [28], similar to the one applied for **3,** established the absolute configuration of Lys as d, while that of the rest of the amino acids as l, assigning structure **4** to anabaenopeptin KB899 (Figure 4).

### 2.4. Structural Elucidation of the New Micropeptins

**Figure 6:**
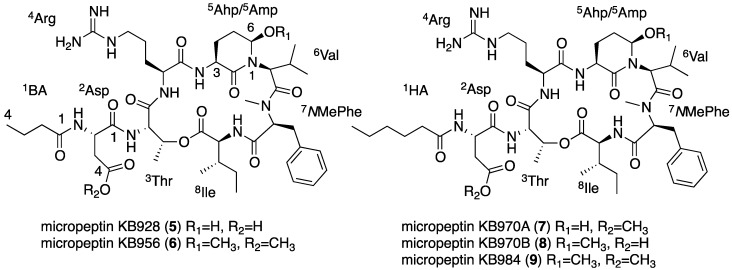
The structures of micropeptins KB928 (**5**), KB956 (**6**), KB970A (**7**), KB970B (**8**) and KB984 (**9**).

Micropeptin KB928 (**5**, Figure 6) was isolated as an amorphous white material that possessed a positive HR ESI MS quasi-molecular ion at *m*/*z* 929.5090 ([M + H]+), corresponding to a molecular formula of C_44_H_69_N_10_O_12_ and 16 degrees of unsaturation. Its NMR data, measured in DMSO-*d*_6_ (Table 3 and Supplementary Table S5), presented signals characteristic of the micropeptin-type cyclic depsipeptide [25], *i.e.*, five secondary amide protons (8.6–7.2 ppm), an ester oxymethine proton (5.45 ppm), an amide methyl group (2.73 ppm), the distinctive hydroxyl signal of a 3-amino-6-hydroxy-2-piperidone (Ahp) moiety (6.11 ppm), nine carboxylic or amide sp^2^ carbons (173–169 ppm) and ester and aminal oxymethine carbons (72.2 and 74.2 ppm, respectively). The interpretation of the COSY and TOCSY NMR data (in DMSO-*d*_6_, Supplementary Table S5) established the sequence of the signals of α-NH-α-H-side chain protons of Asp, *O*-substituted-Thr, Arg, Ahp, Ile, the sequence of the signals of α-H-side chain protons of *N*,*N*-disubstituted-Val and Phe and mono-substituted *n*-propane. Using correlations from the hetero-nuclear HSQC and HMBC experiments (Supplementary Table S5) allowed the expansion of the later fragments to the entire acid residues, except for Arg, whose amide carbon was assigned based on an HMBC correlation of Ahp-NH (7.29 ppm) with the amide carbon (170.3 ppm) and the NOE of the later amide NH with Arg-H-2.

**Table 3:** ^1^H and ^13^C NMR data of Compounds **5**–**9** in DMSO-*d*_6_.

Compound										
	5		6		7		8		9	
Position	δ_C_^a^	δ_H_^b^	δ_C_^a^	δ_H_^b^	δ_C_^a^	δ_H_^b^	δ_C_^c^	δ_H_^d^	δ_C_^a^	δ_H_^b^
^1^BA/^1^HA ^e^										
1	172.0	-	172.8	-	173.0	-	173.0	-	173.1	-
2	37.4	2.11 t	37.3	2.11 t	35.4	2.13 t	35.6	2.13 t	35.7	2.13 t
3	18.9	1.53 qi	18.9	1.55 qi	25.2	1.52 qi	25.1	1.53 qi	25.2	1.51 qi
4	13.8	0.88 t	13.8	0.88 t	31.1	1.22 m	31.1	1.25 m	31.1	1.23 m
5	-	-	-	-	22.1	1.27 m	22.0	1.27 m	22.1	1.27 m
6	-	-	-	-	14.2	0.84 t	14.1	0.85 t	14.2	0.84 t
^2^Asp										
1	172.6	-	171.4	-	171.6	-	171.7	-	171.6	-
2	49.8	4.55 m	49.5	4.86 q	49.7	4.71 ddd	49.7	4.63 m	49.6	4.71 q
3	39.0	2.58 m	35.5	2.80 m	35.7	2.82 dd	35.4	2.80 m	35.4	2.81 m
		2.20 m		2.60 dd		2.58 dd		2.58 m		2.58 dd
4	172.0	-	170.9	-	171.0	-	172.0	-	171.0	-
*N*H	-	8.09 d	-	8.33 d	-	8.27 d	-	8.28 d	-	8.29 d
*O*Me	-	-	51.7	3.56 s	51.8	3.56 s	-	-	51.8	3.56 s
^3^Thr										
1	169.2	-	169.0	-	169.3	-	169.1	-	169.2	-
2	54.8	4.62 d	54.9	4.60 d	55.1	4.57 d	54.9	4.60 d	55.1	4.60 d
3	72.2	5.45 q	72.2	5.49 q	72.2	5.47 q	72.3	5.48 q	72.3	5.49 q
4	17.8	1.17 d	18.2	1.18 d	18.2	1.19 d	17.8	1.17 d	18.0	1.18 d
*N*H	-	7.44 brd	-	7.65 d	-	7.46 d	-	7.63 d	-	7.68 d
^4^Arg										
1	170.3		170.3	-	170.4	-	170.4	-	170.5	-
2	51.8	4.28 m	52.2	4.28 m	52.5	4.26 m	52.2	4.28 m	52.4	4.27 m
3	27.5	2.02 m	27.7	2.02 m	27.6	2.02 m	27.8	2.02 m	27.9	2.02 m
		1.45 m		1.45 m		1.45 m		1.45 m		1.45 m
4	25.2	1.45 m	25.4	1.45 m	25.8	1.45 m	25.4	1.45 m	25.5	1.45 m
5	40.1	3.05 m	40.8	3.08 m	41.0	3.09 m	40.2	3.09 m	40.7	3.09 m
*N*H	-	8.57 d	-	8.57 d	-	8.50 d	-	8.54 d	-	8.56 d
5-*N*H	-	7.49 brt	-	7.53 t	-	7.49 t	-	7.47 m	-	7.56 t
6	156.9	-	156.8	-	157.0	-	156.9	-	157.0	-
6-*N*H,*N*H2	-	7.30 brm	-	7.30 brm	-	7.30 brm		7.30 brm	-	7.30 brm
		6.60 brm		6.70 brm		6.80 brm		6.77 brm		6.85 brm
^5^Ahp/^5^Amp										
2	169.5	-	169.2	-	169.5	-	169.2	-	169.3	-
3	49.0	4.44 m	49.3	4.45 m	49.3	4.44 m	49.4	4.47 m	49.4	4.47 m
4	21.7	2.55 m	21.7	2.40 brq	21.9	2.53 m	21.7	2.40 brq	21.9	2.40 brq
		1.73 m		1.70 m		1.73 m		1.75 m		1.70 m
5	29.2	1.70 m	23.8	2.05 m	30.0	1.72 m	23.8	2.05 m	23.9	2.05 m
				1.75 m				1.70 m		1.75 m
6	74.2	4.91 brs	83.3	4.44 brs	74.3	4.91 brs	83.3	4.44 brs	83.4	4.44 brs
*N*H	-	7.30 d	-	7.23 m	-	7.36 d		7.24 m	-	7.25 m
*O*H/*O*Me		6.11 d	55.5	3.02 s	-	6.17 d	55.6	3.02 s	55.6	3.02 s
^6^Val										
1	169.8	-	169.7	-	170.0	-	169.8	-	169.9	-
2	55.7	4.31 d	55.7	4.35 d	56.1	4.31 d	55.8	4.35 d	55.9	4.36 d
3	27.5	1.90 m	27.2	1.95 m	27.8	1.90 m	27.3	1.95 m	27.4	1.95 m
4	18.2	0.46 d	18.9	0.46 d	18.4	0.46 d	18.3	0.46 d	18.4	0.46 d
5	18.1	−0.20 d	17.8	−0.24 d	18.4	−0.19 d	17.8	−0.23 d	18.0	−0.23 d
^7^*N*MePhe										
1	169.2	-	169.1	-	169.5	-	169.2	-	169.3	-
2	60.6	5.06 brd	60.8	5.09 brd	60.8	5.06 brd	60.9	5.10 brd	61.0	5.10 brd
3	34.3	3.28 m	34.2	3.25 m	34.4	3.26 m	34.3	3.30 m	34.4	3.29 brd
		2.80 m		2.80 m		2.80 dd		2.81 dd		2.80 m
4	137.8		137.7	-	137.9	-	137.8	-	137.8	-
5,5′	129.8	7.23 d	129.7	7.22 m	129.9	7.22 d	129.8	7.22 m	129.9	7.22 m
6,6′	128.7	7.26 t	128.8	7.27 m	128.9	7.27 d	128.9	7.27 m	129.0	7.26 m
7	126.8	7.19 t	126.9	7.19 m	127.0	7.19 d	127.0	7.19 m	127.0	7.19 m
*N*Me	30.3	2.73 s	30.2	2.73 s	30.4	2.73 s	30.3	2.73 s	30.4	2.73 s
^8^Ile										
1	172.7	-	172.6	-	172.9	-	172.6	-	172.7	-
2	55.9	4.69 dd	56.6	4.64 dd	56.0	4.63 dd	55.6	4.64 m	55.8	4.65 dd
3	37.4	1.78 m	37.7	1.74 m	37.3	1.77 m	37.8	1.74 m	37.8	1.74 m
4	24.7	1.23 m	24.8	1.31 m	24.8	1.23 m	24.9	1.30 m	25.0	1.30 m
		1.04 m		1.10 m		1.02 m		1.10 m		1.10 m
5	11.3	0.81 t	11.1	0.83 t	11.4	0.80 t	11.2	0.85 t	11.2	0.84 t
6	16.0	0.83 d	16.0	0.85 d	16.2	0.84 t	16.0	0.87 d	16.1	0.87 d
*N*H	-	7.68 d	-	6.96 d	-	7.76 d	-	6.95 d	-	6.96 d

^a^ One hundred megahertz; ^b^ 400 MHz; ^c^ 125 MHz; ^d^ 500 MHz; ^e^ BA: Butiric acid; HA: Hexanoic acid.

The acid units were combined to the linear structure, ^1^butyric acid (^1^BA)-^2^Asp-^3^Thr-^4^Arg-^5^Ahp-^6^Val-^7^*N*MePhe-^8^Ile, using HMBC correlations between ^2^Asp-NH and ^1^BA-CO, ^3^Thr-NH and ^2^Asp-CO, ^4^Arg-NH and ^3^Thr-CO, ^5^Ahp-NH and ^4^Arg-CO, ^6^Val-2 and ^5^Ahp-CO, ^7^*N*MePhe-*N*Me and ^6^Val-CO and ^8^Ile-NH and ^7^*N*MePhe-CO. The lactone bridge of **5** was established based on the HMBC correlation of ^3^Thr-H-3 with ^8^Ile-CO. The relative configuration of the Ahp was established as 3S*,6R* based on the chemical shifts of the protons and carbons of the Ahp moiety that were found to be identical to those of cyanopeptolin S [20] and the NOE between H-4ax (2.55 m) and the axial 6-OH (6.11 d, 3.2 Hz). Marfey’s analysis [28] of the amino acids derived from the hydrolysis of **5** (with and without oxidation of the aminal) revealed the presence of only l-form Arg, Asp, Glu (from Ahp), Ile, *N*MePhe, Thr and Val. The absolute configuration of the chiral centers of the Ahp moiety were thus established as 3*S*, 6*R*.

Marfey’s analysis does not distinguish between *allo-*Ile and Ile and between *allo-*Thr and Thr. The NMR data was thus used to confirm the identity of these amino acids. We have shown in the past that the proton and carbon chemical shifts of the methyl-groups of Ile in the micropeptins are sensitive to the absolute configuration of C-3 of Ile [36]. Based on this observation and the chemical shifts of the Ile methyl groups (δ_C_ 16.0 and 11.3 for Me-5 and Me-6, respectively) in **5**, it was established as l-Ile. The observed *J*-value (less than 1 Hz) between H-2 and H-3 of the substituted threonine in Compound **5** suggested that, as in the case of all known micropeptins, it should be threonine and not *allo-*threonine [37]. On the basis of these arguments, the structure of micropeptin KB928 was determined as **5**.

**Figure 7:**
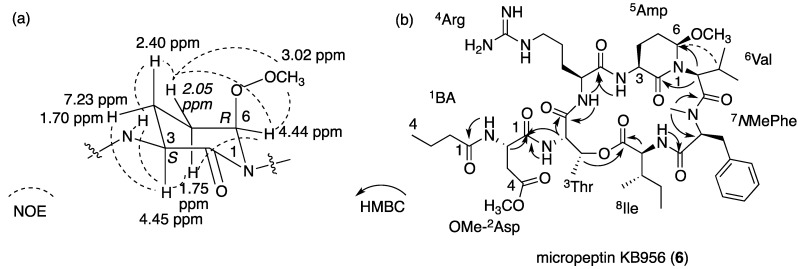
(**a**) NOE correlations that established the relative configuration of the Amp unit of **6**; (**b**) HMBC and NOE correlations that established the amino acid sequence of micropeptin KB956 (**6**).

Micropeptin KB956 (**6**, Figure 6) was isolated as a glassy solid, which exhibited an HR ESI MS protonated-quasi-molecular ion at *m*/*z* 957.5412, corresponding to the molecular formula C_46_H_73_N_10_O_12_ and 16 degrees of unsaturation. Its ^1^H and ^13^C NMR data in DMSO-*d*_6_ (Table 3) were almost identical to those of **5**, except for the appearance of two additional methoxy groups (δ_H_ 3.56 s and 3.02 s and δ_C_ 51.7 and 55.5). A full assignment of the NMR data (Supplementary Table S6) by interpretation of the correlations from homo-nuclear 2D NMR experiments (COSY, TOCSY and ROESY) and hetero-nuclear 2D NMR experiments (HSQC and HMBC) revealed that **6** contained 4-*O*Me-Asp (δ_H_ 3.56 s and δ_C_ 51.7) and 3-amino-6-methoxy-2-piperidone (Amp, δ_H_ 3.02 s and δ_C_ 55.5), instead of Asp and Ahp that exist in **5**. The assignment of the relative configuration of the Amp moiety is presented in Figure 7a, and assembly of the acid units to the cyclic structure is presented in Figure 7b. A similar analysis [28] to the one applied for **5** established the absolute configuration of the acid units in **6** as l-Arg, l-4-*O*Me-Asp, l-Ile, l-*N*MePhe, l-Thr, l-Val and 3*S*, 6*R*-Amp. Based on these arguments, the structure of micropeptin KB956 was established as **6**.

Micropeptin KB970A (**7**, Figure 6), a glassy solid, exhibited an HR ESI MS protonated molecular ion at *m*/*z* 971.5563 corresponding to the molecular formula C_47_H_75_N_10_O_12_ and 16 degrees of unsaturation. The molecular formula of **7** exceeded that of **5** in three methylenes. The ^1^H and ^13^C NMR data of **3** in DMSO-*d*_6_ (Table 3) resembled that of **5**, except for an extra ester methoxy group (δ_H_ 3.56 s and δ_C_ 51.8) and two aliphatic methylenes (δ_H_ 1.27 m and 1.22 m, and δ_C_ 22.1 t and 31.1 t, respectively). The assignment of the NMR data of **7** (Supplementary Table S7) by interpretation of the 2D NMR experiments revealed that **7** contained a 4-*O*Me-Asp (like **6**) and a hexanoic acid instead of Asp and butyric acid in **5**. The absolute configuration of the asymmetric centers was established by a similar procedure as described for **5**. Structure **7** was thus assigned to micropeptin KB970A.

Micropeptin KB970B (**8**, Figure 6) was isolated as a glassy material with a similar HR ESI MS protonated molecular ion, *m*/*z* 971.5561, and an identical molecular formula C_47_H_75_N_10_O_12_ to that of **7**. The ^1^H and ^13^C NMR spectra of **8** (Table 3) were almost identical to those of **7**, except for the chemical shifts of the methoxy moiety (δ_H_ 3.02 s and δ_C_ 55.6) and the amino piperidone moiety. A full assignment of the NMR data of **8** (Supplementary Table S8) revealed that it differs from **7** in having the Amp moiety instead of the Ahp moiety (in **7**) and Asp instead of 4-*O*Me-Asp. The assignment of the absolute configuration of the chiral centers was achieved in a similar way to that of **5**, assigning structure **8** to micropeptin KB970B.

Micropeptin KB984 (**9**, Figure 6) differed in 14 mass unit from **7** and **8**, exhibiting an HR ESI MS quasi-molecular ion at *m/z* 985.5722 and a molecular formula of C_48_H_77_N_10_O_12_ that differed in a CH_2_ from that of **7** and **8**. A comparison of the ^1^H and ^13^C NMR spectra of **9** (in DMSO-*d*_6_, Table 3) with those of **7** and **8** suggested that **9** contained both 4-*O*Me-Asp (δ_H_ 3.56 s and δ_C_ 51.8) and Amp (δ_H_ 3.02 s and δ_C_ 55.6) moieties. The assignment of the 1D and 2D NMR data of **5** (Supplementary Table S9) and the absolute configuration of the chiral centers, as described above for **5**, established the acid units in micropeptin KB984 as l-Arg, l-4-*O*Me-Asp, l-Ile, l-*N*MePhe, l-Thr, l-Val, 3*S*,6*R*-Amp and hexanoic acid and its structure as **9**.

**Figure 8:**
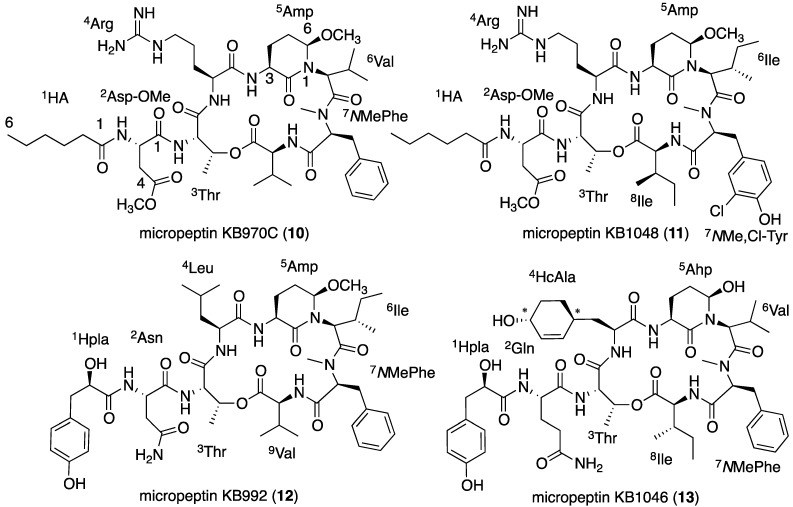
The structures of micropeptins KB970C (**10**), KB1048 (**11**), KB992 (**12**) and KB1046 (**13**).

Micropeptin KB970C (**10**, Figure 8) presented ^1^H and ^13^C NMR spectra (DMSO-*d*_6_) almost identical to those of **9** and an HR ESI MS protonated molecular ion at *m*/z 971.5560, in accordance with the molecular formula C_47_H_75_N_10_O_12_. The major difference between the ^1^H NMR spectra of **10** and **9** (Table 4 and Table 3, respectively) was at the high end of the spectrum, where **10** presented a doublet methyl (δ_H_ 0.85) instead of the triplet methyl of **9** (δ_H_ 0.84). The 14 mass units difference between **10** and **9**, and the one carbon difference in the ^13^C NMR spectra (Table 3 and Table 4) suggested that **6** contained a valine instead of the isoleucine of **9**. A full assignment of the NMR data of **10** (Supplementary Table S10) proved this assumption, while Marfey’s analysis [28], similar to the one used for **5**, established the absolute configuration of all of the chiral centers, as l, ascribing structure **10** to micropeptin KB970C.

**Table 4:** ^1^H and ^13^C NMR data of Compounds **10**–**13** in DMSO-*d*_6_.

Compound									
	10		11			12		13	
Position	δ_C_^a^	δ_H_^b^	δ_C_^a^	δ_H_^b^	Position	δ_C_^a^	δ_H_^b^	δ_C_^c^	δ_H_^d^
^1^HA					^1^Hpla				
1	172.9	-	173.0	-	1	174.2	-	173.6	-
2	35.6	2.13 t	35.5	2.17 t	2	73.0	3.99 ddd	72.7	4.01 ddd
3	25.1	1.52 qi	25.1	1.51 qi	3	41.0	2.93 dd	39.8	2.87 dd
							2.54 m		2.55 m
4	31.0	1.25 m	31.0	1.22 m	4	129.1		128.8	-
5	22.1	1.27 m	22.1	1.24 m	5,5′	130.5	7.02 d	130.4	7.00 d
6	14.1	0.84 t	14.8	0.85 t	6,6′	115.0	6.64 d	115.0	6.62 d
^2^Asp					7	155.8	-	155.8	-
1	171.5	-	171.5	-	2-OH	-	5.60 d	-	5.52 d
2	49.5	4.70 q	49.5	4.70 q	7-OH	-	9.11 s	-	9.06 s
3	35.3	2.80 m	35.5	2.81 dd	^2^Asn/^2^Gln				
		2.58 dd		2.59 dd					
4	170.8	-	170.9	-	1	171.8	-	172.4	-
*N*H	-	8.27 d	-	8.28 d	2	49.3	4.70 q	51.4	4.51 dt
*O*Me	51.7	3.56 s	51.7	3.57 s	3	36.9	2.54 m	28.9	1.82 m
									1.75 m
^3^Thr					4	172.2	-	31.6	2.10 t
1	169.1	-	169.1	-	5			174.0	-
2	55.0	4.58 d	55.0	4.59 d	*N*H	-	8.23 d	-	7.78 d
					*N*H_2_	-	7.39 s6.89 s	-	7.18 s6.70 s
3	72.2	5.50 q	72.3	5.47 q	^3^Thr				
4	17.9	1.18 d	17.8	1.17 d	1	169.2	-	169.6	-
*N*H	-	7.68 d	-	7.71 d	2	55.1	4.60 d	55.3	4.62 d
^4^Arg					3	72.2	5.50 q	71.8	5.52 q
1	170.4	-	170.3	-	4	17.9	1.22 d	17.9	1.23 d
2	52.2	4.27 m	52.1	4.29 m	*N*H	-	7.74 d	-	8.20 d
3	27.8	2.02 m	27.7	2.00 m	^4^Leu/^4^HcAla				
		1.45 m		1.45 m					
4	25.4	1.45 m	25.4	1.45 m	12	171.0	-	170.7	-
5	40.2	3.09 m	40.1	3.08 m	2	51.0	4.28 ddd	50.0	4.33 m
*N*H	-	8.56 d	-	8.56 d	3	39.0	1.80 m	36.6	1.89 m
							1.39 m		1.49 m
5-*N*H	-	7.56 t	-	7.50 t	4	24.4	1.50 m	32.0	1.99 m
6	156.9	-	156.9	-	5	23.9	0.87 d	132.2	5.42 brd
6-*N*H,*N*H_2_	-	7.30 brm	-	7.30 brm	6	21.2	0.78 d	133.0	5.58 brd
		6.90 brm		6.90 brm					
^5^Amp					7			65.5	3.97 m
2	169.2	-	169.3	-	8			31.6	1.80 m
									1.20 m
3	49.4	4.47 m	49.4	4.46 m	9			26.2	1.70 m
									0.96 m
					*N*H		8.45 d	-	8.47 d
					OH				4.63 d
4	21.7	2.40 brq	21.9	2.40 brq					
		1.75 m		1.75 m					
5	23.8	2.05 m	23.9	2.06 brd	^5^Amp/^5^Ahp 2	169.3	-	169.6	-
		1.70 m		1.70 m					
6	83.3	4.44 brs	83.3	4.46 brs	3	49.4	4.47 m	48.9	4.45 m
*N*H	-	7.24 m	-	7.26 d	4	21.9	2.40 q	22.0	2.55 m
							1.70 m		1.72 m
*O*Me	55.6	3.03 s	55.7	3.02 s	5	23.9	2.05 brd	29.9	1.71 m
							1.75 m		
^6^Val/^6^Ile 1	169.8	-	169.9	-	6	83.3	4.43 brs	74.2	4.92 brs
2	55.8	4.36 d	54.1	4.46 m	*N*H	-	7.21 m		7.37 d
3	27.3	1.95 m	33.1	1.86 m	*O*H/*O*Me	55.5	3.01 s		6.09 brs
				1.10 m					
4	18.3	0.46 d	23.9	0.63 m	^6^Ile/^6^Val 1	169.8	-	169.9	-
5	17.9	−0.23 d	10.4	0.63 m	2	54.1	4.43 m	56.0	4.32 d
6	-	-	13.7	−0.13 d	3	32.9	1.78 m	27.7	1.90 m
^7^*N*MePhe 1	169.4	-	169.3	-	4	23.5	1.05 m	18.3	0.46 d
^7^Cl*N*MeTyr							0.59 m		
2	61.0	5.10 brd	61.0	5.08 brd	5	10.5	0.59 m	18.2	−0.21 d
3	34.3	3.30 m	32.9	3.20 brd	6	13.7	−0.30 d		
		2.80 m		2.71 m					
4	137.7	-	129.3		^7^*N*MePhe 1	169.3	-	169.3	-
5(5′)	129.8	7.23 d	130.7	7.13 s	2	60.9	5.17 dd	60.7	5.08 brd
6(6′)	128.9	7.27 t	120.1	-	3	34.4	3.30 m	34.4	3.28 m
							2.80 dd		2.80 m
7	127.0	7.19 t	152.3	-	4	137.8	-	137.8	-
8	-	-	117.0	6.84 d	5,5′	129.8	7.21 d	129.9	7.22 m
9	-	-	129.6	6.96 d	6,6′	128.9	7.25 t	128.8	7.26 m
*N*Me	30.4	2.75 s	30.4	2.71 s	7	126.9	7.19 t	126.7	7.19 m
*O*H	-	-	-	9.96 s	*N*Me	30.5	2.74 s	30.3	2.73 s
^8^Val/^8^Ile 1	172.5	-	172.6	-	^8^Val/^8^Ile 1	172.5	-	172.9	-
2	56.7	4.60 m	54.8	4.79 dd	2	56.5	4.64 dd	55.7	4.77 d
3	31.2	2.00 m	37.6	1.80 m	3	31.4	1.97 m	37.7	1.80 m
4	19.4	0.89 d	26.0	1.32 m	4	19.5	0.83 d	24.7	1.26 m
				1.10 m					1.00 m
5	18.1	0.83 d	11.6	0.91 t	5	18.0	0.77 d	11.5	0.80 t
6	-	-	14.1	0.77 d	6	-	-	16.3	0.83 d
*N*H	-	6.97 d	-	6.80 d	*N*H	-	6.90 d	-	7.67 d

^a^ One hundred twenty five megahertz ; ^b^ 500 MHz; ^c^ 100 MHz; ^d^ 400 MHz.

Micropeptin KB1048 (**11**, Figure 8) was isolated as a glassy solid that exhibited a HR ESI MS complex quasi-molecular ion at *m/z* 1049.5448/1051.5444 (3:1, indicative of one chlorine atom) and a molecular formula of C_49_H_78_ClN_10_O_13_. The ^1^H and ^13^C NMR spectra (DMSO-*d*_6_) of **11** (Table 4) were similar to those of **9** (Table 3) but presented some differences in the aromatic and the aliphatic regions. In the ^1^H NMR spectra, **11** presented a 1,2,4-*tri*-substituted phenol in the aromatic region, and three triplet and two doublet methyl signals at the higher end of the spectrum, while the rest of the spectrum seemed almost identical with that of **9**.

The assignment of the NMR data of **11** (Supplementary Table S11), by interpretation of the correlations from the homo- and hetero-nuclear 2D NMR experiments (COSY, TOCSY, ROESY, HSQC and HMBC), revealed that **11** contained hexanoyl, 4-*O*Me-aspartyl, *O*-substituted threonyl, arginyl, Amp, *N*,*N*-disubstituted isoleucyl, *N*Me-*o*-chlorotyrosyl and isoleucyl moieties. These moieties were assembled to planar structure **11** through HMBC and ROESY correlations. Appling Marfey’s methodology [28] established the absolute configuration of all of the amino acids as of the l-configuration. The observed *J-*value between H-2 and H-3 (0–1 Hz) of the threonine unit of **11** suggested that it should be l-threonine and not l-*allo*-threonine [37]. The 3*S*,6*R* absolute configuration of the Amp moiety was inferred (similar to the one presented in Figure 7a) from the NOEs of axial H-3 with axial H-5 (δ_H_ 1.70), axial H-4 (δ_H_ 2.40 brq, 12.8 Hz) with equatorial H-5 (δ_H_ 2.06 brd, 13.4 Hz) and equatorial H-6 (δ_H_ 4.46) with axial H-5 and equatorial H-5, based on the *S* absolute configuration of Glu that was established by Marfey’s analysis. In the case of the *N*,*N*-disubstituted-Ile, the carbon chemical shifts measured for Compound **11**, 10.4 (C-5) and 13.7 (C-6) ppm, were found to be similar to those measured for l-Ile, 10.3 (C-5) and 13.9 (C-6) ppm (established for nostopeptins A and B by chiral-GCMS [38]), and different from those measured for *allo-*Ile, 12.2 (C-5) and 14.1 (C-6) ppm (established for micropeptin KT1042 [27]). For the Ile moiety at the carboxylic end of the peptide, the measured carbon chemical shifts were 11.6 (C-5) and 14.1 (C-6) ppm, matching those of l-*allo*-Ile (established for oscillapeptin J by chiral-GCMS [39]), 11.4 (C-5) and 14.3 (C-6) ppm and differing from those of l-Ile (established for nostopeptins A and B by chiral-GCMS [38]), 11.3 for (C-5) and 16.1 (C-6) ppm. Based on these arguments, structure **11** was assigned to micropeptin KB1048.

Micropeptin KB992 (**12**, Figure 8) was isolated as a transparent solid that presented an HR ESI MS quasi-molecular ion ([M + Na]^+^) at *m*/*z* 1015.5121 corresponding to the molecular formula C_50_H_72_N_8_NaO_13_ and 19 degrees of unsaturation. Its ^1^H NMR spectrum (DMSO-*d*_6_, Table 4) presented signals corresponding to a mono-substituted phenyl ring, a *para*-substituted phenol, three doublet secondary amide protons (a forth signal was shown to be buried under the signals of the phenyl ring by COSY correlation), two singlet primary-amide protons, eleven protons next to electron withdrawing groups, two singlet methyl groups attached to electronegative atoms and, among others, six doublet methyl groups and one triplet methyl group in the aliphatic region. Among other signals, the ^13^C NMR spectrum (Table 4) presented signals of eight carbonyls, eight aromatic signals in accordance with one mono-substituted phenyl and one *para*-substituted phenol and eleven carbons next to electron withdrawing groups.

The interpretation of the 1D and 2D NMR spectra of **12** (Supplementary Table S12) revealed the planar structures of *p*-hydroxyphenyl lactyl (Hpla), aspaginyl, *O*-substituted threonyl, leucyl, Amp, *N*,*N*-disubstituted isoleucyl, *N*Me-phenylalanyl and isoleucyl, which closed a lactone ring with the oxygen of the threonyl moiety. The absolute configurations of the amino acids (all L) and the Amp moiety (3*S*,6*R*) were assigned for **11** as described above. The absolute configuration of Hpla was assigned as D by chiral-HPLC. On the basis of these arguments, structure **12** was assigned to micropeptin KB992.

Micropeptin KB1046 (**13**, Figure 8) presented an HR ESI MS quasi-molecular ion ([M + Na]^+^) at *m*/*z* 1069.5228, corresponding to the molecular formula C_53_H_74_N_8_NaO_14_ and 21 degrees of unsaturation. The ^1^H and ^13^C NMR spectral data of **13** in DMSO-*d*_6_ (Table 4) shared a significant number of structural elements with **12**, *i.e.*, the primary amide protons, Hpla, *N*Me-Phe, Ile, Thr and Val, while the signals of Amp and Leu (in **12**) were substituted by signals characteristic of Ahp (δ_H_ 6.07 and 4.92, δ_C_ 74.2) and 3-(7-hydroxycyclohex-2-enyl)-alanyl (HcAla) (δ_H_ 5.42, 5.58, 3.97, δ_C_ 132.2, 133.0, 65.5) [40]. The primary amide protons were assigned by the interpretation of the 1D and 2D NMR experiments (Supplementary Table S13) to a Gln moiety, and the structure of the Ahp moiety, including the absolute configuration (3*S*,6*R*), was assigned in a similar fashion to the one described for **5**.

**Figure 9:**
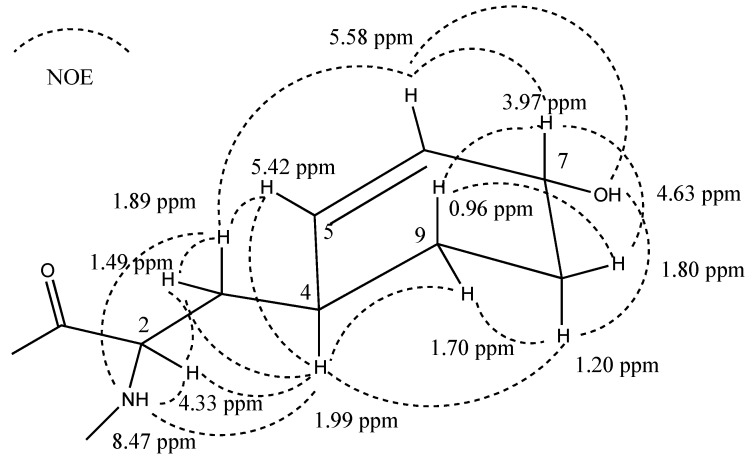
NOE correlations that established the relative configuration of the hydroxycyclohexenyl moiety of **13**.

COSY and TOCSY correlations (Supplementary Table S13) were used to assign the sequence of the proton signals of HcAla: α-NH (δ_H_ 8.47) through H-9_pax_ and H-9_peq_ and of the latter two with H-4, while the HSQC correlations assigned the carbons of this moiety. The carboxyl of HcAla was assigned through HMBC correlation of Ahp-α-NH with the amide carbonyl that resonated at δ_C_ 170.7 and NOE correlations of Ahp-α-NH with HcAla-α-NH and H-2. Assuming a twisted boat conformation for the cyclohexenyl moiety, the pseudoaxial H-8 (H-8_pax_) and H-9 (H-9_pax_) were identified by their shift to a higher field. The NOE correlation of H-9_pax_ with H-7 and of H-8_pax_ with H-4, as well as the rest of the NOEs of this spin system shown in Figure 9 established both as pseudoaxial and the relative configuration of the hydroxyl cyclohexenyl moiety as 4S*,7R*. Although the NOE pattern of H-2, 3a, 3b and 4 pointed to a restricted rotation (Figure 9), it was not possible to assume the relative configuration of Positions 2 and 4. The absolute configuration of C-2 of HcAla was determined by the advanced Marfey method [41]. The hydrolysate of **13** was reacted with l-and d-FDAA, and the retention times of the HcAla-DAA derivatives were obtained using HPLC-MS. The retention time of the HcAla-l-DAA (46.9 min) was shorter than that of HcAla-d-DAA (50.2 min), suggesting that HcAla-C-2 is of the l-configuration [41]. The absolute configuration of the other amino acids of **13** was elucidated by a combination of Marfey’s method [28] and NMR data, as described above for **5**–**12**. Based on these arguments, structure **13** was assigned to micropeptin KB1046.

Micropeptins **5**–**13** could be divided into two separate groups; those that contain a short fatty acid in the side chain and arginine at the fourth position from the amino termini of the peptide and those that contain an Hpla residue at the side chain and lipophilic acid at the fourth position. However, the known micropeptins that were isolated from the bloom material also contained combinations of short fatty acids in the side chains and lipophilic residues at Position 4, and hydroxy acids at the side chain aside from arginine at Position 4. This was more in accordance with the regular varieties observed in bloom-forming cyanobacteria.

### 2.5. Biological Activity Assessment

The biological activity of Compounds **1**–**13** was determined for the inhibition of the proteases, trypsin and chymotrypsin (Table 5). The weak inhibitory activity of aeruginosin KB676 (**1**) can be explained by the steric hindrance of the prenyl substituent, which interferes with the interaction of the guanidine residue of this aeruginosin with the deep acidic binding pocket of trypsin [42]. It is an established phenomenon that micropeptins, which contain arginine or lysine moieties next to their Ahp/Amp residues, are inhibitors of trypsin-type serine proteases [35], explaining the inhibition of trypsin by the arginine containing **5**–**11**. When lipophilic amino acids, such as leucine, tyrosine, glutamine and HcAla, occupied the same position in the micropeptins, chymotrypsin-type serine proteases were usually inhibited [37], explaining the inhibition of chymotrypsin by **12** and **13**. The inhibition of chymotrypsin by **11**, which contained the arginine moiety next to the Amp, was probably a result of the stronger (relative to Phe) interaction of *N*Me-*o*-chlorotyrosine with the enzyme, similar to our previous observation for micropeptins HU909 and HU895A [18].

**Table 5:** Protease inhibition properties of Compounds **1**–**13**.

Compound	Trypsin (IC_50_ in μM)	Chymotrypsin (IC_50_ in μM)
Aeruginosin KB676 (**1**)	40.0	>45.5
Microphycin KB921 (**2**)	>45.5	>45.5
Anabaenopeptin KB906 (**3**)	>45.5	>45.5
Anabaenopeptin KB899 (**4**)	>45.5	>45.5
Micropeptin KB928 (**5**)	0.09	>45.5
Micropeptin KB956 (**6**)	0.62	>45.5
Micropeptin KB970A (**7**)	0.09	>45.5
Micropeptin KB970B (**8**)	0.65	>45.5
Micropeptin KB984 (**9**)	1.12	>45.5
Micropeptin KB970C (**10**)	4.27	>45.5
Micropeptin KB1048 (**11**)	2.01	0.63
Micropeptin KB992 (**12**)	>45.5	0.87
Micropeptin KB1046 (**13**)	>45.5	0.22

### 2.6. Methods of Dereplication

Over the past several years, we have examined several methods to avoid the isolation and structural elucidation of known metabolites from cyanobacterial bloom material. Among the methods we applied were MALDI-TOF MS of on-plate extracts of small amounts of cyanobacteria colonies that gave reproducible results for the major components isolated from the large-scale extract of the bloom material, but failed to give reproducible results for the minor isolated components. In some cases, it seemed as if easily-ionized natural products hindered the ionization of some types of natural products. The yields of the bioactive natural products from cyanobacterial blooms are usually low (3 × 10^−3^% to 1 × 10^−4^%), and these compounds are accompanied by large amounts of fatty-acids and related metabolites that interfere with their chromatography and ionization when LCMS is used for dereplication. In our attempts to use the ESI LCMS methodology for dereplication of cyanobacterial bloom extracts, we found that we are usually able to detect the major bioactive metabolites that are available at yields of about 1 × 10^−3^% of the crude extract, but fail to detect the minor ones. The methodology we currently use combines bioactivity (usually protease inhibition)-guided isolation and purity verification by ESI LCMS and NMR, which allows us to identify the previously known compounds before we start the structural elucidation. This methodology allowed us to identify the stereoisomers of the Choi unit in the aeruginosins [15,27,35] and *allo*-Ile- *versus* Ile-containing anabaenopeptins [15].

## 3. Experimental Section

### 3.1. General Experimental Procedures

Optical rotation values were obtained on a Jasco P-1010 polarimeter at the sodium D line (589 nm; concentrations are reported in g/100 mL. UV spectra were recorded on an Agilent 8453 spectrophotometer (Santa Clara, California, CA, USA). IR spectra were recorded on a Bruker Tensor 27 FT-IR instrument (Billerica, Massachusetts, MA, USA). NMR spectra were recorded on a Bruker DRX-500 spectrometer at 500.13 MHz for ^1^H and 125.76 MHz for ^13^C and a Bruker Avance 400 Spectrometer at 400.13 MHz for ^1^H, 100.62 MHz for ^13^C (Billerica, Massachusetts, MA, USA). DEPT, COSY-45, gTOCSY, gROESY, gHSQC, gHMQC and gHMBC spectra were recorded using standard Bruker pulse sequences. Mass spectra and MS/MS analyses were recorded on a Waters MaldiSynapt instrument (Milford, Massachusetts, MA, USA). HPLC separations were performed on a Jasco HPLC system (Halifax, Nova-Scotia, NS, Canada) (Model PU-2080-plus pump, Model LG-2080-04 Quaternary gradient unit and Model MD-2010-plus Multi-wavelength detector). An Elisa ELx808 reader (Bio-Tek Instruments, Inc (Winooski, VT, USA)) was used for protease inhibition assays.

### 3.2. Biological Material

An assembly of *Microcystis* spp. (Tel Aviv University Collection Number IL-381) was collected in November, 2008, from a commercial fishpond in Kibbutz Kfar Blum, Israel (33°10′45.1′′N; 35°34′37.1′′E). Lyophilized samples of the collected cyanobacteria are deposited at the culture collection of Tel Aviv University.

### 3.3. Isolation Procedure

The freeze-dried cells (IL-381, 950 g) were extracted with 7:3 MeOH:H_2_O (3 × 3 L). The crude extract (142 g) was evaporated to dryness, and aliquots of the extract were fractionated (10 g in each separation) on an octadecyl-silica (ODS) (YMC-GEL, 120A, 4.4 × 6.4 cm) flash column with an increasing concentration of MeOH in H_2_O. The combined fractions from the 14 reversed-phase column separations were tested for inhibition of trypsin and chymotrypsin, and the active fractions (6, 7 and 8) were further purified by the biological activity-guided isolation described below. The combined Fraction 6 (1:1 MeOH:H_2_O, 1.72 g) was subjected to a Sephadex LH-20 column in 7:3 MeOH:H_2_O to obtain thirteen fractions. Fractions 8–9 from the Sephadex LH-20 column were separated on a reversed-phase HPLC (YMC C-8, 5 μm, 250 mm × 20 mm, Diode Array Detector (DAD) at 238 nm, flow rate 5.0 mL/min) in 45:55 0.1% aq. TFA:MeOH to obtain microcystin LR (5.7 mg, retention time (rt) 48.9 min, 6.0 × 10−^4^% yield based on the dry weight of the cells). Combined Fractions 10–12 from the Sephadex LH-20 column were separated on the same reversed-phase HPLC column and with the same solvent system as the previous separation to afford four pure compounds, anabaenopeptin KB899 (**4**; 5.7 mg, rt 48.9 min, 6.0 × 10−^4^% yield) and the known anabaenopeptin 915 (3.4 mg, rt 27.3 min, 3.6 × 10−^4^% yield), anabaenopeptin G (4.4 mg, rt 32.6 min, 4.6 × 10−^4^% yield) and anabaenopeptin MM913 (7.0 mg, rt 60.3 min, 7.4 × 10−^4^% yield). Fraction 2 from the former separation was fractionated again on the same column in 1:1 0.1% aq. TFA:MeOH to obtain aeruginosin 298B (4.4 mg, rt 17.0 min, 4.6 × 10−^4^% yield), anabaenopeptin 908 (5.8 mg, rt 19.5 min, 6.1 × 10−^4^% yield) and anabaenopeptin H (4.0 mg, rt 21.0 min, 4.2 × 10−^4^% yield).

The combined Fraction 7 from the initial reversed phase (RP) separation (6:4 MeOH:H_2_O, 1.83 g) was subjected to a Sephadex LH-20 column in 1:1 MeOH:CHCl_3_ to obtain thirteen fractions, of which Fractions 2–3 and 4–6 were further separated. The combined Fractions 2 and 3 from the latter separation were fractionated again on a Sephadex LH-20 column in 7:3 MeOH:H_2_O to obtain twelve fractions. The combined Fractions 6 and 7 from the former separation were separated on a reversed-phase HPLC (YMC C-8, 5 μm, 250 mm × 20 mm, DAD at 238 nm, flow rate 5.0 mL/min) in 1:3 0.1% aq. TFA:MeOH to obtain the known anabaenopeptin HU892 (5.2 mg, rt 19.0 min, 5.5 × 10^−4^% yield). Fraction 5 from the former HPLC was re-chromatographed on the same column in 1:1 0.1% aq. TFA:MeOH to obtain the known micropeptin LH920 (1.9 mg, rt 23.5 min, 2.0 × 10^−4^% yield) and micropeptin KB928 (**5**; 2.6 mg, rt 22.7 min, 2.7 × 10^−4^% yield). Fractions 4 to 6 from the Sephadex LH-20 column were re-separated on the same column eluted in 7:3 MeOH:H_2_O to obtain twelve fractions. Fractions 3–6 contain the pure known oscillamide C (2.1 mg, 2.2 × 10^−4^% yield), while the combined Fractions 7–9 (184.8 mg) were further separated on a reversed-phase HPLC (YMC C-8, 5 μm, 250 mm × 20 mm, DAD at 238 nm, flow rate 5.0 mL/min) in 2:3 0.1% aq. TFA:MeOH to obtain aeruginosin DA495A (2.6 mg, rt 17.1 min, 2.7 × 10^−4^% yield), anabaenopeptin KB906 (**3**; 4.2 mg, rt 21.4 min, 4.4 × 10^−4^% yield), micropeptin KB1046 (**13**; 4.8 mg, rt 33.5 min, 5.0 × 10^−4^% yield) and ichytopeptin A (2.6 mg, rt 35.4 min, 2.7 × 10^−4^% yield), as well as Fractions 6 and 9, which were further separated by HPLC. Fraction 6 was separated on the YMC C-8 column to yield cyanopeptolin SS (1.2 mg, rt 21.1 min, 1.2 × 10^−4^% yield), cyanopeptolin S (1.5 mg, rt 29.1 min, 1.6 × 10^−4^% yield) and micropeptin LH1020 (1.8 mg, rt 23.7 min, 1.9 × 10^−4^% yield), while Fraction 9 was separated on the same column in 3:7 0.1% aq. TFA:MeOH to obtain micropeptin HM978 (1.2 mg, rt 25.8 min, 1.2 × 10^−4^% yield) and micropeptin KB992 (**12**; 2.1 mg, rt 31.9 min, 2.2 × 10^−4^% yield).

The combined Fraction 8, from the initial RP separation (6:4 MeOH:H_2_O, 1.70 g), was subjected to a Sephadex LH-20 column in 1:1 MeOH:CHCl_3_ to obtain eleven fractions of which Fractions 4–8 were separated again on the same column eluted in 7:3 MeOH:H_2_O to obtain twelve fractions. Fractions 9–12 from the latter column were fractionated on the YMC C-8 column in 1:4 0.1% aq. TFA:MeOH to furnish the known aeruginazole DA1304 (1.0 mg, rt 30.9 min, 1.1 × 10^−4^% yield). Fractions 5–7 from the former Sephadex LH-20 column were separated again on the same column eluted in 1:1 MeOH:H_2_O to obtain ten fractions out of which, combined Fractions 5–8 and Fraction 9 were further separated on the C-8 HPLC column in 1:3 0.1% aq. TFA:MeOH. Fraction 9 yielded micropeptin KB1048 (**11**; 2.8 mg, rt 21.5 min, 2.9 × 10^−4^% yield), while Fractions 5–8 yielded micropeptin KB956 (**6**; 5.0 mg, rt 18.5 min, 5.3 × 10^−4^% yield), micropeptin KB984 (**9**; 9.6 mg, rt 25.9 min, 1.0 × 10^−3^% yield) and Fraction 3, which was separated on the same column in 1:1 0.1% aq. TFA:MeOH to afford micropeptin KB970A (**7**; 3.5 mg, rt 13.7 min, 3.7 × 10^−4^% yield), micropeptin KB970B (**8**; 2.1 mg, rt 14.5 min, 2.2 × 10^−4^% yield) and micropeptin KB970C (**10**; 0.9 mg, rt 15.4 min, 0.9 × 10^−4^% yield). Combined fractions 8–10 from the second Sephadex LH-20 column were separated again on the same column eluted in 1:1 MeOH:H_2_O to obtain twelve fractions, out of which combined Fractions 3–5 and 6–8 were further separated on the YMC C-8 HPLC column in 2:3 0.1% aq. TFA:acetonitrile. Fractions 3–5 yielded aeruginosin KB676 (**1**; 3.5 mg, rt 23.1 min, 3.7 × 10^−4^% yield), while Fractions 6–8 yielded microphycin KB921 (**2**; 3.3 mg, rt 40.8 min, 3.5 × 10^−4^% yield) and aeruginazole A (8.5 mg, rt 36.2 min, 8.9 × 10−4% yield).

Aeruginosin KB676 (**1**): Amorphous white solid; [α]_D_^23^ −14° (*c* 0.17, MeOH); UV (MeOH) λ_max_ (log ε) 202 (4.29), 224 (3.79), 278 (3.12) nm; IR (ATR Diamond) ν_max_ 2936, 1648, 1542, 1202, 1137 cm^−1^; ^1^H and ^13^C NMR (Table 1 and Supplementary Table S1); HR MALDI-TOF MS *m*/*z* 677.4031 [M + H]^+^, (calcd. for C_37_H_53_N_6_O_6_, 677.4027). Retention times of amino acid (AA) Marfey’s derivatives: d-Phe 52.9 min (l-Phe 50.1 min), (2*S*,3a*S*,6*S*,7a*S*)-Choi (l-6-*epi*Choi) 41.5 min, ((2*R*,3a*R*,6*R*,7a*R*)-Choi (d-3a,7a-*diepi*Choi)) 38.3 min and ((2*S*,3a*S*,6*R*,7a*S*)-Choi (l-Choi)) 42.0 min. Retention time of d-hydroxyphenyllactic acid (Hpla) on the chiral-phase column 5.69 min (l-Hpla 5.41 min).

Microphycin KB921 (**2**): Amorphous white solid; [α]_D_^23^ −36° (*c* 0.19, MeOH); UV (MeOH) λ_max_ (log ε) 202 (4.25), 208 (4.15), 217 (3.94) nm; IR (ATR Diamond) ν_max_ 2934, 1671, 1653, 1542, 1203, 1138 cm^−1^; ^1^H and ^13^C NMR (Table 2 and Supplementary Table S2); HR MALDI-TOF MS *m*/*z* 944.4650 [M + Na]^+^, (calcd. for C_49_H_63_N_9_NaO_9_, 944.4646). Retention times of AA Marfey’s derivatives: l-Glu 36.8 min (d-Glu 37.7 min), l-Pro 41.2 min (d-Pro 49.0 min), 2 × l-Ala 45.4 min (d-Ala 49.0 min), 3 × l-Phe 50.0 min (d-Phe 52.9 min), l-Leu 50.0 min (d-Leu 53.9 min).

Anabaenopeptin KB906 (**3**): Amorphous white solid; [α]_D_^23^ −62° (*c* 0.13, MeOH); UV (MeOH) λ_max_ (log ε) 202 (4.18), 224 (3.67), 278 (2.91) nm; IR (ATR Diamond) ν_max_ 3422, 1664, 1206 cm^−1^; ^1^H and ^13^C NMR (Table 2 and Supplementary Table S3); HR MALDI-TOF MS *m*/*z* 907.5417 [M + H]^+^, (calcd. for C_46_H_71_N_10_O_9_, 907.5405). Retention times of AA Marfey’s derivatives: l-Arg 29.6 min (d-Arg 29.4 min), l-*N*MeHty 39.6 min, 2 × l-Ile 43.1 min (d-Ile 46.2 min), d-Lys 45.2 min (l-Lys 44.6 min), l-Hph 46.8 min (d-Hph 49.4 min).

Anabaenopeptin KB899 (**4**): Amorphous white solid; [α]_D_^23^ −45° (*c* 0.38, MeOH); UV (MeOH) λ_max_ (log ε) 202 (4.34), 225 (3.97), 278 (3.40) nm; IR (ATR Diamond) ν_max_ 3310, 2934, 1638, 1542, 1204 cm^−1^; ^1^H and ^13^C NMR (Table 2 and Supplementary Table S4); HR MALDI-TOF MS *m*/*z* 922.4693 [M + Na]^+^, (calcd. for C_48_H_65_N_7_NaO_10_, 922.4691). Retention times of AA Marfey’s derivatives: l-Val 44.4 min (d-Val 47.9 min), l-*N*MeHty 46.4 min, l-Ile 48.1 min (d-Ile 50.7 min), d-Lys 49.0 min (l-Lys 50.46 min), l-Hph 52.0 min (d-Hph 55.2 min), l-Tyr 54.5 min (d-Tyr 57.1 min).

Micropeptin KB928 (**5**): Amorphous white solid; [α]_D_^23^ −82° (*c* 0.17, MeOH); UV (MeOH) λ_max_ (log ε) 202 (4.37), 217 (4.00) nm; IR (ATR Diamond) ν_max_ 3367, 2966, 1650, 1542, 1204, 1138 cm^−1^; ^1^H and ^13^C NMR (Table 3 and Supplementary Table S5); HR MALDI-TOF MS *m*/*z* 929.5090 [M + H]^+^, (calcd. for C_44_H_69_N_10_O_12_, 929.5096). Retention times of AA Marfey’s derivatives: l-Arg 28.2 min (d-Arg 26.9 min), l-Asp 33.0 min (d-Asp 34.0 min), l-Thr 32.7 min (d-Thr 35.9 min), l-Glu 34.6 min (d-Glu 35.9 min), l-Val 43.5 min (d-Val 47.6 min), l-Ile 47.3 min (d-Ile 52.1 min), l-*N*MePhe 48.2 min.

Micropeptin KB956 (**6**): Amorphous white solid; [α]_D_^23^ −128° (*c* 0.13, MeOH); UV (MeOH) λ_max_ (log ε) 202 (4.34), 217 (3.84) nm; IR (ATR Diamond) ν_max_ 3367, 2962, 1639, 1527, 1200, 1177, 1133 cm^−1^; ^1^H and ^13^C NMR (Table 3 and Supplementary Table S6); HR MALDI-TOF MS *m*/*z* 957.5412 [M + H]^+^, (calcd. for C_46_H_73_N_10_O_12_, 957.5409). Retention times of AA Marfey’s derivatives: l-Arg 27.8 min (d-Arg 26.6 min), l-Asp 28.6 min (d-Asp 29.5 min), l-Thr 27.8 min (d-Thr 31.2 min), l-Glu 30.1 min (d-Glu 31.2 min), l-Val 37.8 min (d-Val 41.8 min), l-Ile 41.4 min (d-Ile 45.2 min), l-*N*MePhe 42.5 min.

Micropeptin KB970A (**7**): Amorphous white solid; [α]_D_^23^ −54° (*c* 0.23, MeOH); UV (MeOH) λ_max_ (log ε) 202 (4.21), 217 (3.57) nm; IR (ATR Diamond) ν_max_ 3366, 2964, 1653, 1541, 1203, 1136 cm^−1^; ^1^H and ^13^C NMR (Table 3 and Supplementary Table S7); HR MALDI-TOF MS *m/z* 971.5563 [M + H]^+^, (calcd. for C_47_H_75_N_10_O_12_, 971.5566). Retention times of AA Marfey’s derivatives: l-Arg 28.5 min (d-Arg 27.3 min), l-Asp 29.3 min (d-Asp 30.3 min), l-Thr 28.5 min (d-Thr 32.0 min), l-Glu 31.1 min (d-Glu 31.7 min), l-Val 38.3 min (d-Val 45.6 min), l-Ile 41.8 min (d-Ile 45.2 min), l-*N*MePhe 42.8 min.

Micropeptin KB970B (**8**): Amorphous white solid; [α]_D_^23^ −88° (*c* 0.14, MeOH); UV (MeOH) λ_max_ (log ε) 202 (4.30), 217 (3.74) nm; IR (ATR Diamond) ν_max_ 3366, 2933, 1652, 1557, 1202, 1135 cm^−1^; ^1^H and ^13^C NMR (Table 3 and Supplementary Table S8); HR MALDI-TOF MS *m*/*z* 971.5561 [M + H]^+^, (calcd. for C_47_H_75_N_10_O_12_, 971.5566). Retention times of AA Marfey’s derivatives: l-Arg 28.5 min (d-Arg 27.3 min), l-Asp 29.2 min (d-Asp 30.2 min), l-Thr 28.5 min (d-Thr 31.9 min), l-Glu 31.0 min (d-Glu 32.0 min), l-Val 38.3 min (d-Val 42.7 min), l-Ile 41.8 min (d-Ile 45.7 min), l-*N*MePhe 42.8 min.

Micropeptin KB984 (**9**): Amorphous white solid; [α]_D_^23^ −62° (*c* 0.17, MeOH); UV (MeOH) λ_max_ (log ε) 202 (4.32), 217 (3.74) nm; IR (ATR Diamond) ν_max_ 3367, 2962, 1638, 1542, 1200, 1178 cm^−1^; ^1^H and ^13^C NMR (Table 3 and Supplementary Table S9); HR MALDI-TOF MS *m*/*z* 985.5732 [M + H]^+^, (calcd. for C_48_H_77_N_10_O_12_, 985.5736). Retention times of AA Marfey’s derivatives: l-Arg 28.1 min (d-Arg 26.9 min), l-Asp 28.8 min (d-Asp 29.8 min), l-Thr 28.1 min (d-Thr 31.5 min), l-Glu 30.6 min (d-Glu 31.7 min), l-Val 37.8 min (d-Val 41.8 min), l-Ile 41.4 min (d-Ile 45.2 min), l-*N*MePhe 42.4 min.

Micropeptin KB970C (**10**): Amorphous white solid; [α]_D_^23^ −87° (*c* 0.06, MeOH); UV (MeOH) λ_max_ (log ε) 202 (4.37), 217 (4.00) nm; IR (ATR Diamond) ν_max_ 3432, 2922, 1645, 1208 cm^−1^; ^1^H and ^13^C NMR (Table 4 and Supplementary Table S10); HR MALDI-TOF MS *m*/*z* 971.5560 [M + H]^+^, (calcd. for C_47_H_75_N_10_O_12_, 971.5566). Retention times of AA Marfey’s derivatives: l-Arg 28.2 min (d-Arg 27.5 min), l-Asp 34.1 min (d-Asp 34.8 min), l-Thr 33.2 min (d-Thr 36.3 min), l-Glu 34.9 min (d-Glu 36.3 min), 2 × l-Val 43.8 min (d-Val 48.0 min), l-*N*MePhe 48.4 min.

Micropeptin KB1048 (**11**): Amorphous white solid; [α]_D_^23^ −11° (*c* 0.19, MeOH); UV (MeOH) λ_max_ (log ε) 202 (4.49), 230 (3.67), 282 (2.91) nm; IR (ATR Diamond) ν_max_ 3366, 2962, 1652, 1541, 1201, 1136 cm^−1^; ^1^H and ^13^C NMR (Table 4 and Supplementary Table S11); HR MALDI-TOF MS *m/z* 1,049.5448/1,051.5444 (3:1) [M + H]^+^, (calcd. for C_49_H_78_^35^ClN_10_O_13_, 1049.5438). Retention times of AA Marfey’s derivatives: l-Arg 28.7 min (d-Arg 27.5 min), l-Asp 29.4 min (d-Asp 30.4 min), l-Thr 28.7 min (d-Thr 32.7 min), l-Glu 31.3 min (d-Glu 32.3 min), 2 × l-Ile 42.0 min (d-Ile 46.6 min), l-*N*MeClTyr 49.9 min.

Micropeptin KB992 (**12**): Amorphous white solid; [α]_D_^23^ −47° (*c* 0.14, MeOH); UV (MeOH) λ_max_ (log ε) 202 (4.29), 208 (4.14), 278 (2.92) nm; IR (ATR Diamond) ν_max_ 3421, 2931, 1648, 1542 cm^−1^; ^1^H and ^13^C NMR (Table 4 and Supplementary Table S12); HR MALDI-TOF MS *m/z* 1,015.5121 [M + Na]^+^, (calcd. for C_50_H_72_N_8_NaO_13_, 1015.5117). Retention times of AA Marfey’s derivatives: l-Asp 33.9 min (d-Asp 34.6 min), l-Thr 33.3 min (d-Thr 36.3 min), l-Glu 35.5 min (d-Glu 36.5 min), l-Val 43.9 min (d-Val 47.8 min), l-Ile 47.6 min (d-Ile 51.7 min), l-*N*MePhe 48.3 min, l-Leu 48.5 min (d-Leu 2.3 min). Retention time of d-hydroxyphenyllactic acid (Hpla) on the chiral-phase column 5.43 min (l-Hpla 5.27 min).

Micropeptin KB1046 (**13**): Amorphous white solid; [α]_D_^23^ −36° (*c* 0.39, MeOH); UV (MeOH) λ_max_ (log ε) 202 (5.39), 225 (3.90), 276 (3.29) nm; IR (ATR Diamond) ν_max_ 3367, 2933, 1638, 1533, 1202 cm^−1^; ^1^H and ^13^C NMR (Table 4 and Supplementary Table S13); HR MALDI-TOF MS *m/z* 1,069.5228 [M + Na]^+^, (calcd. for C_53_H_74_N_8_NaO_13_, 1069.5222). Retention times of AA Marfey’s derivatives: l-Thr 28.8 min (d-Thr 32.2 min), 2 × l-Glu 31.4 min (d-Glu 32.2 min), l-Val 38.6 min (d-Val 43.0 min), l-Ile 42.2 min (d-Ile 46.0 min), l-*N*MePhe 43.2 min. Retention time of HcAla derivatives with l-FDAA (46.9 min) and d-FDAA (50.2 min). Retention time of d-hydroxyphenyllactic acid (Hpla) on the chiral-phase column 5.36 min (l-Hpla 5.24 min).

### 3.4. Determination of the Absolute Configuration of the Amino Acids by Marfey’s Method

Compounds **1**–**13** (0.3 mg each) were hydrolyzed in 6 N HCl (1 mL). The reaction mixture was maintained in a sealed glass bomb at 104 °C for 16 h. The acid was removed *in vacuo*, and the residue was re-suspended in 250 μL of H_2_O. FDAA solution ((1-fluoro-2,4-dinitrophenyl)-5-l-alanine amide) or ((1-fluoro-2,4-dinitrophenyl)-5-d-Alanine amide) in acetone (115 μL, 0.03 M) and NaHCO_3_ (120 μL, 1 M) were added to each reaction vessel. The reaction mixture was stirred at 40 °C for 2 h. Then, HCl (2 M, 60 μL) was added to each reaction vessel, and the solution was evaporated *in vacuo*. The FDAA-amino acids derivatives from hydrolysate were dissolved in 1 mL CH3CN and compared with standard FDAA-amino acids by an HPLC analysis: Hibar LiChrospher 60, RP-select B (5 μm, 250 × 4.6 mm), flow rate 1 mL/min, UV detection at 340 nm, linear gradient elution from 1:9 CH_3_CN:0.1% aq. TFA buffer (pH 3) to CH_3_CN, within 60 min. The absolute configuration of each amino acid was determined by spiking the derivatized hydrolysates with d,l-mixture of the standard derivatized amino acids. As with the advanced Marfey method [41], the retention times of the d- and l-derivatives were compared to determine the configuration of the constituent amino acid.

### 3.5. Determination of the Absolute Configuration of Hydroxy Phenyl Lactic Acid

The extraction of the acid hydrolysates of Compounds **8**, **9** and **13** with ethyl ether separated Hpla from the amino acid salts. The ether was removed *in vacuo*, and the residue was dissolved in MeOH (1 mL). The MeOH solution was analyzed on an Astec, Chirobiotic T, LC stationary phase, 250 × 4.6 mm flow rate 1 mL/min, UV detection at 277 nm, linear elution with 19:1 MeOH:1% aq. triethylammonium acetate buffer (pH 4). The Hpla derivatives from **1**, **12** and **13** were compared with an authentic standard of d,l-Hpla.

### 3.6. Protease Inhibition Assays

The procedures used to determine the inhibitory activity of the new compounds on trypsin and chymotrypsin were described in a previous paper [19]. The range of concentrations used in the assays was between 45.5 μM and 0.011 μM.

## 4. Conclusions

*Microcystis* blooms usually produce a large variety of short peptides, of which the micropeptins are dominant in quantity and diversity. In the current research, the 7:3 MeOH:H_2_O extract yielded 31 different peptides of six structural groups. As in the case of many other *Microcystis* blooms that we have investigated [18,19,24,25], the diversity of the micropeptins (144 isolated variants excluding this publication) isolated was the greatest (15 compounds). The number of the anabaenopeptins (57 isolated variants excluding this publication) was second to the micropeptins, with nine closely-related analogs, while the aeruginosins (the second most diverse group of protease inhibitors in cyanobacterial blooms, 69 isolated variants excluding this publication) were represented by only three analogs, one of which was the prenylated aeruginosin KB676 (**1**). These three groups of modified peptides are biosynthesized by non-ribosomal peptide synthetases that are flexible and capable of synthesizing series of analogues metabolites. The reason(s) for the biosynthesis of these metabolites and their high variability in cyanobacterial water blooms are intriguing, and our current research is aimed at revealing the purpose for the biosynthesis of these metabolites.

## Supplementary Information

1D (^1^H, ^13^C) and 2D NMR (HSQC, HMBC, COSY, ROESY) spectra and HR MS data of Compounds **1**–**13**, tables of full NMR data of **1**–**13** and two figures with the structures of the known metabolites isolated in this study are available.
